# Systemic Periodontal Risk Score Using an Innovative Machine Learning Strategy: An Observational Study

**DOI:** 10.3390/jpm12020217

**Published:** 2022-02-04

**Authors:** Paul Monsarrat, David Bernard, Mathieu Marty, Chiara Cecchin-Albertoni, Emmanuel Doumard, Laure Gez, Julien Aligon, Jean-Noël Vergnes, Louis Casteilla, Philippe Kemoun

**Affiliations:** 1 RESTORE Research Center, Université de Toulouse, INSERM, CNRS, EFS, ENVT, Université P. Sabatier, 31100 Toulouse, France; david2.bernard@inserm.fr (D.B.); chiara.cecchin@protonmail.com (C.C.-A.); emmanuel.doumard@inserm.fr (E.D.); louis.casteilla@inserm.fr (L.C.); philippe.kemoun@univ-tlse3.fr (P.K.); 2 Artificial and Natural Intelligence Toulouse Institute ANITI, 31013 Toulouse, France; 3 Dental Faculty and CHU de Toulouse—Toulouse Institute of Oral Medicine and Science, 31062 Toulouse, France; mathieu.marty@univ-tlse3.fr (M.M.); laure.gez@univ-tlse3.fr (L.G.); jean-noel.vergnes@univ-tlse3.fr (J.-N.V.); 4 Institute of Research in Informatics (IRIT) of Toulouse, CNRS—UMR5505, 31062 Toulouse, France; julien.aligon@ut-capitole.fr; 5 CERPOP, UMR1295 (Axe MAINTAIN), Université P. Sabatier, 31000 Toulouse, France; 6 Population Oral Health Research Cluster of the McGill Faculty of Dental Medicine and Oral Health Sciences, Montreal, QC H3A 1G1, Canada

**Keywords:** personalized oral medicine, machine learning, risk factors, periodontitis

## Abstract

Early diagnosis is crucial for individuals who are susceptible to tooth-supporting tissue diseases (e.g., periodontitis) that may lead to tooth loss, so as to prevent systemic implications and maintain quality of life. The aim of this study was to propose a personalized explainable machine learning algorithm, solely based on non-invasive predictors that can easily be collected in a clinic, to identify subjects at risk of developing periodontal diseases. To this end, the individual data and periodontal health of 532 subjects was assessed. A machine learning pipeline combining a feature selection step, multilayer perceptron, and SHapley Additive exPlanations (SHAP) explainability, was used to build the algorithm. The prediction scores for healthy periodontium and periodontitis gave final F1-scores of 0.74 and 0.68, respectively, while gingival inflammation was harder to predict (F1-score of 0.32). Age, body mass index, smoking habits, systemic pathologies, diet, alcohol, educational level, and hormonal status were found to be the most contributive variables for periodontal health prediction. The algorithm clearly shows different risk profiles before and after 35 years of age and suggests transition ages in the predisposition to developing gingival inflammation or periodontitis. This innovative approach to systemic periodontal disease risk profiles, combining both ML and up-to-date explainability algorithms, paves the way for new periodontal health prediction strategies.

## 1. Introduction

A total of 50% of people over 50 years of age present periodontitis, and they have a potential risk of losing teeth during their lifetimes [1]. Indeed, periodontitis is a chronic inflammatory disease of the tooth-supporting tissues, both the gingiva and the underlying tissues anchoring the tooth root in its surrounding alveolar bone. It results in clinical gingival inflammation and alveolar bone loss with subsequent increasing gingival crevices, typically leading to the formation of periodontal pockets [2]. The shift from periodontal health to periodontitis occurs through a transient gingival inflammation stage (with no underlying root anchorage defect) associated with a dysbiosis [2]. Such periodontal dysbiosis arises from the disruption of gingival host–microbiota homeostasis, a physiological mechanism that serves to prevent the emergence of pathogenic microbiota through appropriate periodontal host defenses, despite the continuous stresses occurring in gingiva throughout a lifespan. Because the mere presence of periodontal pathogens is not sufficient at inducing a dysfunctional clinical phenotype [3], it is currently accepted that the evolution towards periodontitis through host–microbiota homeostasis disruption and gingival inflammation occurs only in susceptible hosts [2,4,5], with an increase in the risk factors associated with periodontal disease. Indeed, susceptibility to periodontitis, as for other inflammatory diseases, appears to change in response to complex interactions between genetic and acquired environmental factors throughout a lifespan (e.g., smoking, pathologies, psychic stress, pregnancy, gender, ethnicity) [6]. These modifiable and non-modifiable risk factors, however, may impact the initiation, progression, and severity of periodontal disease [3,4]. To control periodontitis and its systemic implications, therapies must be introduced as early as possible. Thus, the identification of risk factor profiles for periodontal diseases represents a great challenge to improve periodontal prevention. Some periodontal risk prediction strategies are routinely used, such as Lang and Tonetti’s [7] periodontal risk assessment (PRA). This estimates the risk of susceptibility for periodontal disease progression by a clinical assessment of periodontal lesions, together with the patient’s age, an evaluation of the systemic conditions and, finally, an evaluation of environmental and behavioral factors, such as smoking. The PRA is used for treatment planning and prognosis [8] but requires an oral medicine practitioner for the periodontal assessment. Furthermore, PRA does not consider the complexity of the potential interactions between the different risk factors, including medical and psycho–sociodemographic status, which is critical to target periodontal-susceptible subjects at an early stage, even before the oral symptomatology becomes identifiable by a practitioner [3,5]. In terms of personalized medicine, these multiple interaction assessments are crucial toward implementing individualized prevention and therapeutic strategies. The rise of artificial intelligence (AI), including machine learning (ML), provides exciting opportunities to extract valuable information from complex data to benefit patients [9]. ML strategies seem to be particularly pertinent to predict the factors influencing periodontitis occurrence [10,11]. Despite their undeniable efficacy for prediction, these approaches are often considered as black boxes, with limited explainability. However, the recent development of explainability technologies now offers the possibility of understanding the prediction mechanisms of ML models [12]. The aim of this study was to propose a predictive machine learning algorithm to identify the subjects at risk of developing periodontal diseases, solely based on non-invasive predictors that can easily be collected in the clinic. This innovative approach of a systemic periodontal disease risk score, combining both ML and up-to-date explainability algorithms, paves the way for a new strategy of periodontal health prediction.

## 2. Materials and Methods

This observational study was reported in accordance with the STROBE guidelines [13].

### 2.1. Study Design and Subjects

This observational study was conducted at the Oral Medicine Department of the Toulouse University Hospital Centre (France) during routine visits by three independent and calibrated experts. All patients attending a consultation were considered. To be eligible, the patients and/or guardians needed to understand French and to provide their consent for the data collection and clinical examination. If the oral clinical exam could not be performed, mostly because of lack of cooperation, the patient was excluded. All patients gave their consent. The personal and medical data were collected and computer-processed to analyze the results of this research.

### 2.2. Clinical Procedures

The clinical examinations were conducted by four trained practitioners (P.M., M.M., C.C.A., P.K.), specialists in oral medicine and periodontology, calibrated before the start of the study. The Community Periodontal Index of Treatment Needs (CPITN) score (range 0 to 4) was used to assess periodontal health [14]. The highest CPITN score was considered using partial recordings [15]—CPITN 0: healthy periodontium, CPITN 1: presence of gum bleeding, 2: presence of calculus and gingival bleeding, 3: presence of shallow periodontal pocket (4–5 mm), and 4: presence of deep periodontal pocket (6 mm and above) [14]. As such, CPITN was classified as 0 (healthy periodontium), CPITN 1–2 (gingival inflammation), and CPITN 3–4 (periodontitis). The examinations were conducted at a dental setting with a suitable dental probe, mirror, and light source. The data collection was completed with information obtained by a patient interview (all of the requested information is detailed in Supplementary Table S1) on putative periodontal risk factors i.e., general medical status (presence of a systemic pathology, long-term medicinal treatments), stress (using 0–10 EVA score), socioeconomic status/conditions, and dietary habits. All data were collected anonymously.

### 2.3. Data Visualization, Modeling, and Explanation

Different analysis strategies were successively combined to produce the final periodontal health prediction model.

**Figure 1:**
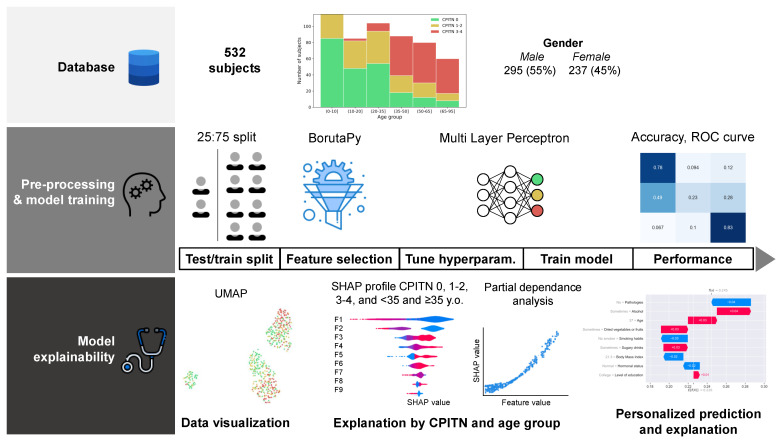
Machine learning analysis pipeline. The analysis pipeline involves three successive steps: (1) constitution of the database with sociodemographic, general medical status, stress, and dietary habits from 532 subjects, together with the periodontal health condition (CPITN); (2) development of the prediction algorithm and evaluation of its performance; (3) model explainability, based on cluster-based visualization of data, SHapley Additive exPlanations (SHAP) profile at the global and individual levels.

The machine learning pipeline (Figure 1) involved: (1) encoding binary and ordinal variables followed by a feature selection step, (2) a random training/test dataset splitting of 75:25, (3) a min–max data scaler followed by a multilayer perceptron model [16], and (4) explainability of the prediction results on the whole dataset (Figure 1). The scikit-learn library v0.24.0 was used as a general framework [17]. BorutaPy v0.3 [18] is a feature selection method able to select a minimal set of features (i.e., variables) that carry significant information for the prediction model. The following hyperparameters were used: 500 estimators, maximal depth of 3, and entropy as a criterion. The profiles of the subjects, with respect to the variables selected by BorutaPy, were visualized by projecting them through UMAP (i.e., uniform manifold approximation and projection [19]), followed by a DBSCAN algorithm using Euclidean distance to identify clusters of subjects. Descriptive statistics were then produced to characterize each cluster.

After min–max normalization, the Boruta-reduced dataset was passed through a multilayer perceptron algorithm (from the scikit-learn library). Since this technique has some hyperparameters and we were seeking the best performing model with minimum overtraining, we explored the hyperparameter space using scikit-optimize v0.8.1. The best combination was retained: four hidden layers with 4, 128, 256, and 8 neurons, respectively, an Adam solver, an ’identity’ activation function, 0.7 beta1 and 0.4 beta2 scores. To assess the performance of the model, we conducted a five-fold cross-validation, and compared the performance values (weighted F1-score [20]) between the training and validation sets. Finally, the complete performance of the model was assessed on the test set (precision, recall/sensitivity, specificity, weighted F1-score, and ROC curve) for each category to predict (CPITN 0, CPITN 1–2, and CPITN 3–4).

The main obstacle to understanding most machine learning models is the “black box” aspect. Once a model has been trained, it is necessary to know the influences and interactions of the attributes behind the classification performed. Kernel SHAP is a model-agnostic method to approximate SHAP values [12]. This method can explain the influence of each attribute of the dataset on the output of the predictive model.

## 3. Results

### 3.1. Description of the Study Population

A total of 532 subjects were examined between 02/01/2019 and 01/03/2021. The mean age of the total sample was 33 ± 15 years (range 2 to 83) with 45% of females (Table S1). The distributions of the subjects’ periodontal health scores by age group are detailed in Figure S1. The maximum proportion of the healthy periodontium was found in the 0–10-year-old group. The prevalence of gingival inflammation (i.e., CPITN score 1–2) increased up until 35 years-old (20%, 40%, and 45% for the 0–10-, 10–20-, and 20–35-year-old groups, respectively) then decreased sharply after 35 years as periodontitis (CPITN score 3–4) increased.

The missing data were encoded by assigning a “−1” value. By mapping all of the variables in two dimensions using an UMAP methodology, three distinct clusters were highlighted according to the sociodemographic characteristics and other risk factors of the individuals (Figure S2). Cluster 1 included the smallest (and mainly contained) children (0–10 years-old), while clusters 2 and 3 consisted of female and male adults, respectively. Within each cluster, a distinction can be drawn between CPITN 0 and CPITN 3–4 (the two groups can be separated on the vertical axis of UMAP), while no distinction can be made for CPITN 1–2. The clusters of adults showed similar value distributions for BMI, smoking habits, systemic pathologies, alcohol, and sugary drinks consumption.

### 3.2. Feature Selection

**Figure 2:**
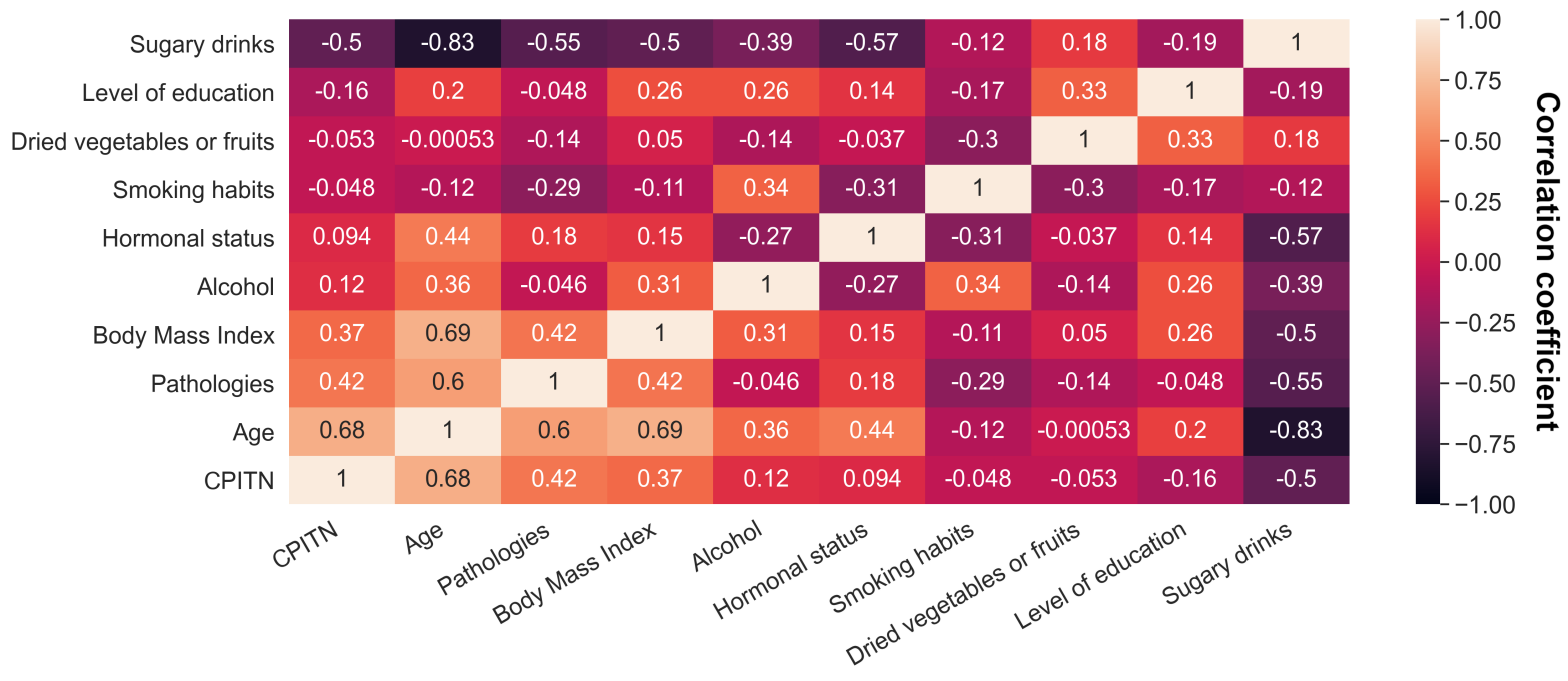
Variable correlation matrix. The matrix shows the Pearson correlation between CPITN and the variables selected by the BorutaPy algorithm. Age, body mass index, and the presence of a systemic disease are positively associated with CPITN, while sugary drink consumption is negatively associated with CPITN. The low association of CPITN with hormonal status can be explained as a multi–variable effect, as the hormonal status depends, among others, on both gender and age.

BorutaPy is a feature selection algorithm designed to select only the relevant variables according to the CPITN group score, thus maintaining a minimum number of explanatory variables to establish the final model. By using the BorutaPy algorithm introduced in Section 2.3, 9 out of 30 (30%) variables were retained, namely age, body mass index (BMI), systemic pathologies, educational level, hormonal status, as well as smoking and nutritional habits, such as consumption of dried vegetables or fruits, sugary drinks, and alcohol. Interestingly, gender, stress, oral hygiene practices, and dental attendance were not sufficiently contributive to be selected by the method. The raw correlation matrix showed that age, BMI, and systemic pathologies were highly positively associated to CPITN, while sugary drink consumption was highly negatively associated to CPITN (Figure 2). Smoking habits and hormonal status did not associate with CPITN, although the BorutaPy algorithm showed that they were needed to predict CPITN. Moreover, many parameters were associated with each other, illustrating the complex interactions between the factors themselves, such as pathologies and smoking habits (r = −0.29) or alcohol and smoking habits (r = 0.34). Building a machine learning model will thus make it possible to capture the complex relationships between the variables.

### 3.3. Data Modeling by Machine Learning Models

**Figure 3:**
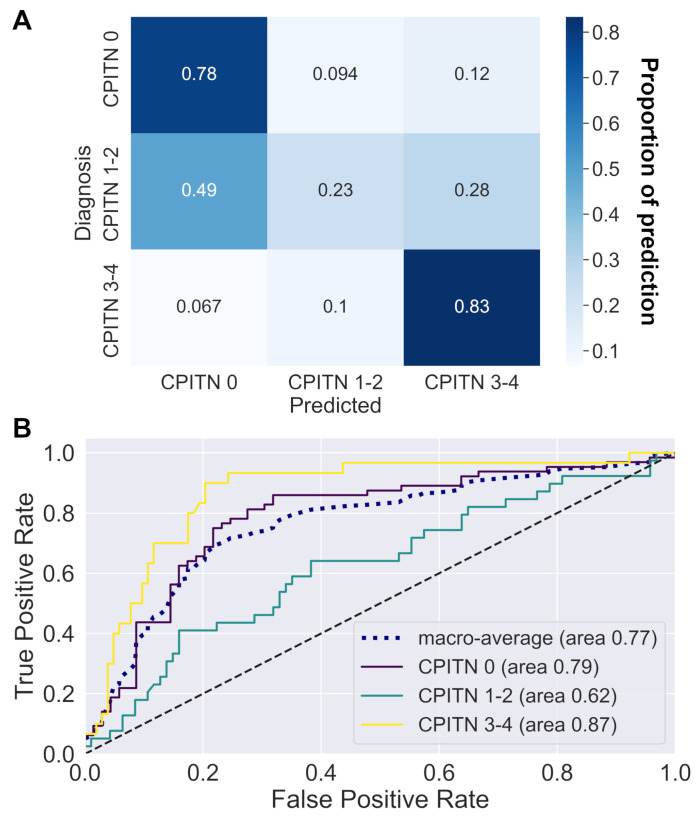
Machine learning model performance. Assessment of the best performing model based on variable selection (BorutaPy), data augmentation, and multilayer perceptron. (**A**) The corresponding confusion matrix indicates the proportion of good predictions for each category of CPITN. The complexity of predicting CPITN 1–2 is, thus, highlighted. (**B**) The ROC curves show the relationship between the true positive rate (sensitivity) and the false positive rate (1 - specificity) for each CPITN category.

The machine learning pipeline consisted of a min–max scaler followed by a multi-layer perceptron step, whose parameters had been tuned on the training data set. Using a five–fold cross-validation, the weighted F1–scores obtained for the training and validation datasets were 0.60 ± 0.03 and 0.57 ± 0.08, respectively. The confusion matrix (Figure 3A) showed good prediction scores for healthy periodontium and periodontitis, but it highlighted some problems in the model for accurate prediction of gingival inflammation (final F1–score of 0.74, 0.32, 0.68, and 0.60 for CPITN 0, CPITN 1–2, CPITN 3–4 and average, respectively). The evaluation metrics presented in Supplementary Table S2 and the ROC curve in Figure 3B illustrate the specificity and sensitivity of the model prediction regarding each group prediction.

**Figure 4:**
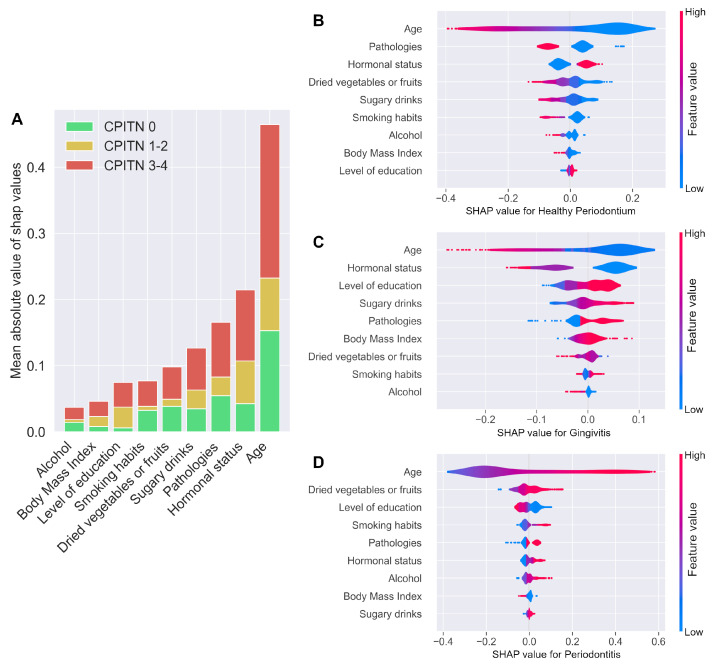
Explanations of the ML model. The “KernelSHAP method” was used for prediction, assigning each attribute (i.e., each variable of the final ML model) with an importance value (SHAP value) for the prediction of specific CPITN scores. (**A**) Variable importance for each CPITN score, sorted by decreasing mean absolute values of SHAP values. SHAP values, according to feature values for subjects with healthy periodontium (**B**), gingival inflammation (**C**), or periodontitis (**D**). The color of each violin plot encodes the value of the associated variable—red for higher values for the variable and blue for lower values. For the categorical variables, a low value can also signify a missing value. For hormonal status, the values in increasing order were as follows: being a man, non–menopausal woman, postmenopausal woman. On the x–axis, a positive SHAP value signifies that the variable, contributes positively to the risk prediction, whereas a negative SHAP value signifies that the variable contributes negatively to the prediction. Variables are shown from the top to the bottom, in order of importance (mean of absolute SHAP values).

The “kernelSHAP method” was used to interpret the predictions, assigning each attribute (i.e., each variable of the final ML model) with an importance value (SHAP value) for a given CPITN score prediction (Figure 4A–D). Age, systemic pathologies (mostly cardiovascular, endocrine, and metabolic diseases), hormonal status, dried vegetable or fruit consumption, and sugary drink consumption were the five most contributive variables used to predict periodontal health, in contrast to body mass index for example (Figure 4A,B). Increased age, dried vegetable or fruit consumption, smoking, and pathologies tended to increase the risk of periodontitis (CPITN 3–4), in contrast to other variables, such as level of education (Figure 4D). Figure 4C shows that age, hormonal status (for women), level of education, sugary drink consumption and pathology tend to increase the risk of gingival inflammation.

**Figure 5:**
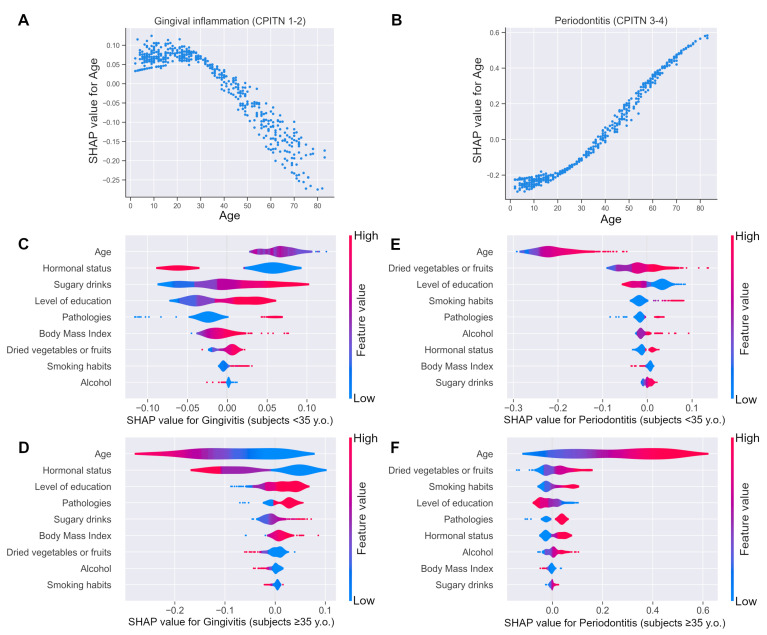
SHAP contribution for CPITN prediction according to the pivotal age of 35 years. (**A**,**B**) The partial dependence plots show how the SHAP values partially depend on the input variables of interest. (**A**) Rise of gingival inflammation risk until 35 years old and decrease thereafter. (**B**) Sigmoid-type relationship of periodontitis risk with age with a sharp transition around 35 years old. (**C**–**F**) SHAP values of gingival inflammation (**C**,**D**) or periodontitis (**E**,**F**) risk according to feature values for subjects < 35 years old (**C**,**E**), and subjects > 35 years old (**D**,**F**). The color of each violin plot encodes the value of the associated variable, red for higher values of the variable and blue for lower values. For the categorical variables, a low value can also signify a missing value. For hormonal status, the values are in increasing order as follows: being a man or a girl (<12 years old), non-menopausal woman, postmenopausal woman. On the x–axis, a positive SHAP value signifies that the variable contributes positively to the risk prediction, whereas a negative SHAP value signifies that the variable negatively contributes to the prediction. Variables are shown from the top to the bottom in order of importance (mean of absolute SHAP values).

The partial dependence plots show how the SHAP values partially depend on the input variables of interest. The model clearly demonstrates the rise in gingival inflammation risk up until 35 years old, and the decrease thereafter (Figure 5A). The SHAP contribution of age for the CPITN 3–4 prediction increased in a sigmoid-type relationship with a sharp transition around 35 years old (Figure 5B). Since 35 years old seemed to be an important transition phase, the explanations were split according to this age. While age is the most important factor for gingivitis prediction, the explainability profile differs between before and after 35 years of age (Figure 5C,D). It is interesting to note that age remains the preponderant factor in predicting periodontitis risk, and that the explanatory profile is quite similar before and after 35 years of age, according to the importance ranking of the variables and the distribution of the SHAP values (Figure 5E,F). When comparing prediction explainability between gingival inflammation and periodontitis, variable importance and ranking are nevertheless not superimposable (Figure 5C–F).

**Figure 6:**
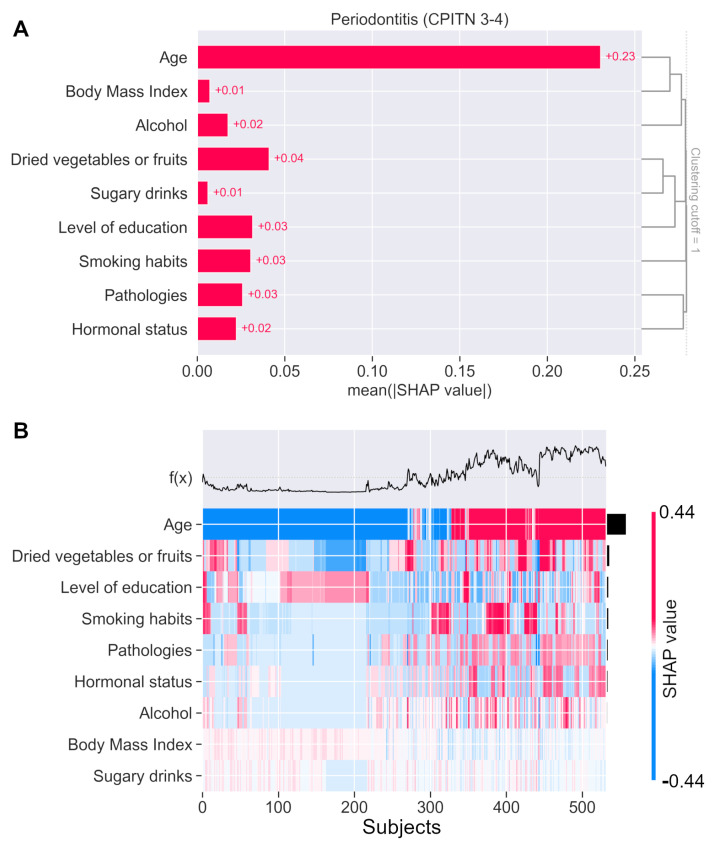
SHAP values correlation and clustering on periodontitis prediction. (**A**) SHAP bar plot clustering (right side) displays the redundancy structure as a dendrogram. Age mainly correlates with BMI and alcohol consumption, while pathologies are strongly associated with hormonal status. Diet seems to be rather dependent on the level of education. (**B**) The SHAP values clustering on the whole population highlights the different risk factor combinations that may explain the prediction of a periodontitis diagnosis. *f(x)* is the predicted probability of periodontitis. Each variable’s SHAP contribution on periodontitis prediction is represented in color by subject.

Interestingly, the analysis of the SHAP values for periodontitis prediction show that age correlates with BMI and alcohol consumption, while diet is more dependent on the level of education, and gender (displayed by hormonal status) is associated with general pathologies (Figure 6A). Moreover, the SHAP values clustering on the whole population highlights that, at the individual level, there is an increase in combinations among age and diet, education, smoking, alcohol consumption, hormonal status, and/or systemic pathologies to explain the probability of a periodontitis diagnosis (Figure 6B).

**Figure 7:**
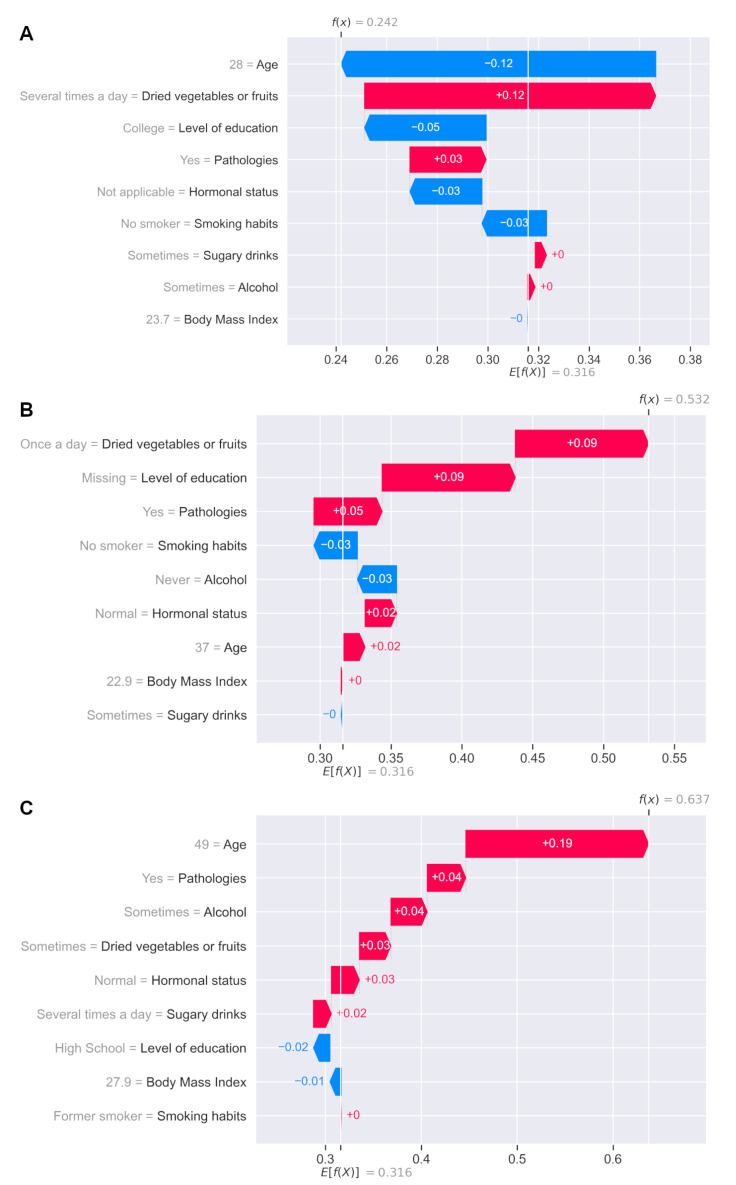
Explanations at the individual level (individual risk prediction). (**A**) A 28-year-old healthy subject predicted to have a 0.24 probability of periodontitis. (**B**) A 37-year-old healthy subject with a 0.53 probability of periodontitis. (**C**) A 49-year-old subject presenting periodontitis-accumulating risk factors and predicted to have a high risk of periodontitis (0.64).

The explanations can also be analyzed at the individual level (individual risk prediction). Figure 7 provides an example of a 28-year-old healthy subject predicted to have a 24% risk of periodontitis. The algorithm interprets the high consumption of dried fruits or vegetables and the existence of a pathology as an increased risk of periodontitis, while age, not smoking, or being male (hormonal status not applicable) are interpreted as decreased risks of periodontitis (Figure 7A). Figure 7B illustrates a 37-year-old woman with no periodontitis but predicted to have a 53% risk of developing this pathology. Figure 7C illustrates a 49-year-old patient with periodontitis-accumulating risk factors, predicted to have a high risk of periodontitis.

## 4. Discussion

This study conducted a machine learning analysis based on an innovative strategy using a wide range of medical and sociodemographic parameters. The results support the hypothesis that, like many age-related inflammatory chronic diseases, periodontitis can be associated with a systemic risk profile, with no reference to oral stressors (e.g., poor hygiene). Conversely, this kind of predictive pattern was not identified for gingival inflammation. This could be explained by the absence in the database of specific oral hygiene variables (e.g., plaque index to highlight a putative poor hygiene) able to differentiate dental plaque-induced gingival inflammations from those preceding periodontitis in susceptible hosts, which are largely uncorrelated with oral hygiene [21].

All variables selected by the feature selection algorithm—i.e., age, systemic pathologies, smoking, and female hormonal status—are well-known risk factors for chronic inflammatory diseases.

Age is the most critical periodontal risk factor. Indeed, our data confirm that most patients over 50 years old display periodontal lesions and that the time span from 35 to 50 years of age is critical for individuals at risk of periodontal disease. In addition, following a trend for increasing life expectancy, periodontitis prevalence is expected to rise, with significant consequences on health, given the bidirectional relationships between periodontal diseases and general pathologies [22]. Unlike the systemic and psycho-sociodemographic risk factors, the biological mechanisms of aging on the pathophysiology of the periodontium are still poorly understood. However, new hypotheses are emerging concerning physiological—tissue health-related—adaptations to the accumulation of stressors over time [23]. The increased prevalence of periodontitis after 50 years of age can be explained by the notion of reserve depletion (or “allostatic load”) as a result of stressors to the oral cavity [23,24,25]. Indeed, early and continuous stressors trigger a set of physiological learning mechanisms—called “allostatic”—to maintain the functions of the periodontium, starting at tooth eruption. One explanation for why this mechanism becomes a disease is that the patient has “drained” his/her ability to adequately respond to repeated stimuli [25,26]. Additionally, allostatic load has been cited as the origin of cardiovascular, metabolic, and even degenerative diseases [27]. On a pathophysiological level, it can also explain the immune depletion and reduced potential for cell renewal and differentiation and, therefore, the disruption in the balance between the host and his/her periodontal microbiota [25]. The effect of life course on periodontal health can be considered an accumulation of stresses over time with variable intensities, each with a probability of impacting the periodontal pathophysiology. Since progressive periodontal tissue exhaustion is an essential prerequisite for the installation of periodontitis [3,26], this implies that structural–functional periodontium alterations begin to set in slowly, several years before the diagnosis, at around the age of 50. It is therefore possible that the 35–50 age transition, highlighted by the partial dependence plot of the contribution of age to periodontitis risk (i.e., the partial dependence plot of SHAP values for age according to age), corresponds to a population displaying the accumulation of stressor-induced periodontal alterations at a subclinical scale before these turn into clinically detectable periodontitis. Merging image acquisition and analysis, biological data (such as proteomics or transcriptomics) and bio–psycho–social data, together with an ML-based analysis strategy, could help physicians to detect the infra-clinical periodontal alterations that precede the emergence of periodontitis.

Interestingly, the ML model showed the presence of a systemic disease (such as chronic inflammatory diseases, e.g., obesity, diabetes, cardiovascular diseases, and metabolic syndrome) to be strongly associated with periodontal health deterioration. Indeed, these conditions are characterized by low-grade inflammation [4,28,29] and were previously found to be associated with periodontal disease [28,30,31]. In obesity, the visceral adipose tissues secrete inflammatory markers (e.g., cytokines, adipokines) inducing increased systemic inflammation and oxidative stress disorders, with an enhancement of the host immune response in the periodontal tissues [28,30,31]. Furthermore, obesity phenotypes are characterized by reduced bacterial species richness and an increase in some periodontal pathogens [32,33]. In the same way, type 2 diabetes is associated with an increased expression of inflammatory cytokines in periodontal tissues and increased inflammation [4], with a reduction in oral microbial diversity and a surge of periodontal pathogens [34,35,36]. Furthermore, epidemiological studies have highlighted an association between periodontitis and cardiovascular diseases (coronary heart disease, cerebrovascular disease, peripheral arterial disease, rheumatic and congenital heart diseases, and venous thromboembolism) [10,37,38], both explained by the deleterious effects of oxidative stress [39,40,41]. Moreover, metabolic syndrome, combining both cardiovascular diseases and obesity [42], may facilitate a pro-oxidant state, potentially decreasing the antioxidant capacity of the periodontal tissues [43]. Overall, it was recently shown that the systemic dysimmunity resulting from metabolism disorders contributes to sustained periodontium inflammation [44].

Smoking, one of the most important periodontal risk factors used by our algorithm, is shown to increase the development and progression of periodontal diseases [4,45], with peripheral vasoconstriction, dysfunction of neutrophils and T cells, production of proinflammatory cytokines, increased permeability of the airway mucosa, and changes in the airway epithelial barrier function [46,47]. Moreover, the proliferation, chemotaxis and attachment of periodontal stromal progenitors are inhibited by nicotine [29]. Smoking also selects specific periodontal pathogens, including Porphyromonas gingivalis, Treponema denticola, and Tannerella forsythia [4]. While gender, per se, was not shown to be an explanatory factor of the algorithm, ascertaining a woman’s hormonal status is important to maintain algorithm accuracy. There is substantial evidence to demonstrate that sex steroid levels greatly influence periodontal health. Indeed, sex hormones are fundamental to skeletal development, vascularization, bone homeostasis, and immune function, including cytokine production [6,48,49]. Age-associated reductions in sex steroids provide insight into the increased susceptibility to periodontitis and alveolar bone loss, particularly among women [48].

Surprisingly, perceived stress is not a key contributor for the prediction model. Although it has been suggested that stress influences periodontitis occurrence, its role in this disease pathophysiology remains debated [50,51]. We do not know whether it impairs the host response at a purely physiological level, causing, for example, impaction of the inflammation pathways, or whether stressed individuals have behavioral traits that induce higher levels of periodontal risk (e.g., smoking, diet, oral hygiene) [52]. It is also possible that stress is hidden by or intertwined with other stress-related variables, such as socioeconomic background [50,53].

As correlation matrix shows, there are multiple interrelationships between the different risk factors for periodontitis. The model considers the interactions between the different factors. However, one must be careful not to infer causality. Future investigations on animal models and the use of recent algorithms dedicated to causality will help to understand the etiopathogenesis of periodontal diseases.

Although a broad recruitment was carried out, the population was drawn from a single hospital recruitment center, whose population may differ slightly from a population received in private practice.

## 5. Conclusions

The onset of periodontitis was shown to be influenced by multiple factors in an interwoven and heterogeneous fashion, making it far from being deterministic. As such, the development of numerical tools capable of predicting the probability of periodontitis offers significant insights in the personalized medicine context. Unlike previous models, the proposed machine learning approach provides a risk score for periodontitis based on individual features, without calling for local or intra-oral factors. Subjects prone to periodontitis could be detected using artificial intelligence by responding to a complex spectrum of determinants combining biological, clinical, and sociodemographic factors. Implemented within the care path, this algorithm could reinforce the diagnostic arsenal of practitioners to identify patients at risk of periodontal disease, paving the way for targeted prevention strategies.

## Data Availability

Data available on request from the authors.

## Supplementary Materials

The following are available online at https://www.mdpi.com/article/10.3390/jpm12020217/s1, Figure S1: Description of the study population: periodontal health distribution by age, Figure S2: UMAP clustering of the subjects’ data, Table S1: Medical and sociodemographic characteristics of the study population, Table S2: performance of the Multilayer Perceptron model for each category of periodontal health to predict.

## Abbreviations

The following abbreviations are used in this manuscript:

AI	artificial intelligence
Body Mass Index	BMI
CNIL	French National Commission for Informatics and Liberties
CPITN	Community Periodontal Index of Treatment Needs
ML	machine learning
MLP	multilayer perceptron
PRA	periodontal risk assessment
SHAP	SHapley Additive exPlanations
