# Supplementary material for: Systemic Periodontal Risk Score Using an Innovative Machine Learning Strategy: An Observational Study
Source: J Pers Med. 2022 Feb 4;12(2):217. doi: 10.3390/jpm12020217 (PMC8879877; doi:10.3390/jpm12020217)
Supplement: Supplementary file 1 [file jpm-12-00217-s001.zip › Table S1.pdf]

Table S1. Medical and sociodemographic characteristics of the study population

	All<br>N = 532	CPITN 0<br>N = 225	CPITN 1-2<br>N = 152	CPITN 3-4<br>N = 155	p-value
**Age**	33.0 ± 22.6	20.7 ± 17.6	28.9 ± 19.5	54.8 ± 14.7	< 0.001
**Sex**	532	225	152	155	0.06
Male	295 (55%)	133 (59%)	72 (47%)	90 (58%)	
Female	237 (45%)	92 (41%)	80 (53%)	65 (42%)	
**BMI**	22.7 ± 5.8	20.5 ± 5.3	22.8 ± 5.9	25.8 ± 4.8	< 0.001
**Smoking habits**	530	225	152	153	< 0.001
Non smoker	379 (72%)	192 (85%)	112 (73%)	75 (49%)	
Former smoker (>10 years)	23 (4%)	2 (1%)	4 (3%)	17 (11%)	
Former smoker (>2 years)	20 (4%)	1 (.5%)	3 (2%)	16 (10%)	
Former smoker (>6 months)	18 (3%)	1 (.5%)	6 (4%)	11 (7%)	
Current smoker	90 (17%)	29 (13%)	27 (18%)	34 (23%)	
**Systemic pathology**	519	218	150	151	< 0.001
No	318 (61%)	172 (79%)	96 (64%)	50 (33%)	
Yes	201 (39%)	46 (21%)	54 (36%)	101 (67%)	
**Level of education**	439	172	124	141	< 0.001
Primary School	78 (18%)	53 (31%)	17 (14%)	8 (6%)	
Technical	67 (15%)	10 (6%)	16 (13%)	40 (28%)	
High School	126 (29%)	48 (28%)	41 (33%)	41 (29%)	
College	168 (38%)	61 (35%)	50 (40%)	52 (37%)	
**Allergies**	465	181	136	148	< 0.001
No	339 (73%)	134 (74%)	98 (72%)	107 (72%)	
Yes	126 (27%)	47 (26%)	38 (28%)	41 (28%)	
**Contraceptive pill**	217	79	50	83	< 0.001
No	143 (66%)	48 (61%)	30 (60%)	63 (76%)	
Yes	74 (34%)	31 (39%)	20 (40%)	20 (24%)	
**Any previous pathological pregnancies**	233	88	55	90	< 0.001
No	204 (88%)	75 (85%)	46 (84%)	83 (92%)	
Yes	29 (12%)	13 (15%)	9 (16%)	7 (8%)	
**Hormonal status**	214	81	48	80	< 0.001
Non-menopausal	140 (65%)	65 (80%)	38 (79%)	32 (40%)	
Menopausal	74 (37%)	16 (20%)	10 (21%)	48 (60%)	
**Perceived stress level (0 to 10)**	4.2 ± 2.4	4.0 ± 2.2	3.9 ± 2.4	4.7 ± 2.5	0.05
**Bread, rusks, or cereals consumption**	529	224	152	153	0.09
Never	23 (4%)	7 (3%)	6 (4%)	10 (7%)	
Sometimes	56 (11%)	28 (13%)	19 (13%)	9 (6%)	
Several times a week	60 (12%)	26 (12%)	20 (13%)	14 (9%)	
Once a day	177 (33%)	84 (38%)	47 (31%)	46 (30%)	
Several times a day	213 (40%)	79 (34%)	60 (39%)	74 (48%)	
**Rice, pasta, semolina, or potatoes consumption**	475	184	139	152	< 0.001
Never	3 (1%)	2 (1%)	0 (0%)	1 (1%)	
Sometimes	41 (9%)	16 (9%)	5 (4%)	20 (13%)	
Several times a week	223 (46%)	90 (49%)	63 (45%)	70 (46%)	
Once a day	138 (29%)	49 (26%)	47 (34%)	42 (28%)	

Several times a day	70 (15%)	27 (15%)	24 (17%)	19 (12%)	
**Dried vegetable or fruit consumption**	475	184	138	153	
Never	33 (7%)	15 (8%)	15 (11%)	3 (2%)	
Sometimes	206 (43%)	87 (47%)	56 (41%)	63 (41%)	
Several times a week	147 (31%)	55 (30%)	44 (32%)	48 (31%)	< 0.001
Once a day	62 (13%)	22 (12%)	16 (11%)	24 (16%)	
Several times a day	27 (6%)	5 (3%)	7 (5%)	15 (10%)	
**Vegetable consumption**	526	223	152	153	
Never	10 (2%)	5 (2%)	4 (2%)	1 (1%)	
Sometimes	53 (10%)	25 (11%)	19 (13%)	9 (6%)	
Several times a week	139 (27%)	56 (25%)	45 (30%)	40 (26%)	0.40
Once a day	160 (30%)	68 (31%)	39 (25%)	53 (35%)	
Several times a day	164 (31%)	69 (31%)	45 (30%)	50 (32%)	
**Fruit consumption**	529	224	152	153	
Never	13 (3%)	8 (4%)	3 (2%)	2 (1%)	
Sometimes	54 (10%)	9 (4%)	18 (12%)	27 (18%)	
Several times a week	123 (23%)	49 (22%)	39 (26%)	35 (23%)	0.001
Once a day	162 (31%)	66 (29%)	49 (32%)	47 (31%)	
Several times a day	177 (33%)	92 (41%)	43 (28%)	42 (27%)	
**Dairy product consumption**	527	224	152	153	
Never	12 (2%)	2 (1%)	3 (2%)	7 (5%)	
Sometimes	55 (10%)	16 (7%)	20 (13%)	21 (15%)	
Several times a week	105 (20%)	44 (20%)	30 (20%)	31 (20%)	0.11
Once a day	167 (32%)	71 (32%)	49 (32%)	47 (30%)	
Several times a day	188 (36%)	91 (40%)	50 (33%)	47 (30%)	
**Meat and egg consumption**	475	184	139	152	
Never	5 (1%)	3 (2%)	1 (1%)	1 (1%)	
Sometimes	39 (8%)	12 (6%)	15 (11%)	12 (8%)	
Several times a week	208 (44%)	86 (47%)	53 (38%)	69 (45%)	< 0.001
Once a day	160 (34%)	58 (31%)	48 (35%)	54 (36%)	
Several times a day	63 (13%)	25 (14%)	22 (15%)	16 (10%)	
**Fish or any other sea-food product consumption**	476	184	139	153	
Never	25 (5%)	13 (7%)	8 (6%)	4 (2%)	
Sometimes	186 (39%)	77 (42%)	59 (42%)	50 (33%)	
Several times a week	210 (44%)	79 (43%)	56 (40%)	75 (49%)	< 0.001
Once a day	46 (10%)	11 (6%)	14 (10%)	21 (14%)	
Several times a day	9 (2%)	4 (2%)	2 (2%)	3 (2%)	
**Ready meal consumption**	529	224	152	153	
Never	157 (30%)	51 (23%)	49 (32%)	57 (37%)	
Sometimes	260 (49%)	125 (56%)	73 (48%)	62 (40%)	
Several times a week	73 (14%)	32 (14%)	14 (9%)	27 (18%)	0.01
Once a day	31 (6%)	14 (6%)	13 (9%)	4 (3%)	
Several times a day	8 (1%)	2 (1%)	3 (2%)	3 (2%)	
**Sugary meal consumption**	518	223	152	153	
Never	26 (5%)	7 (3%)	8 (5%)	11 (7%)	
Sometimes	170 (33%)	55 (25%)	49 (32%)	66 (43%)	
Several times a week	147 (28%)	66 (30%)	33 (22%)	48 (32%)	< 0.001
Once a day	104(20%)	61 (27%)	29 (19%)	14 (9%)	
Several times a day	71 (14%)	34 (15%)	33 (22%)	14 (9%)	
**Appetizer cookie, crisps, or snack cookie consumption**	476	184	139	153	
Never	67 (14%)	17 (9%)	18 (13%)	32 (21%)	< 0.001
Sometimes	277 (58%)	106 (58%)	87 (62%)	84 (55%)	

Several times a week	94 (20%)	44 (24%)	25 (18%)	25 (16%)	
Once a day	21 (4%)	9 (5%)	5 (4%)	7 (5%)	
Several times a day	17 (4%)	8 (4%)	4 (3%)	5 (3%)	
**Food supplements and/or vitamin tablets**	475	184	138	153	
Never	334 (70%)	125 (68%)	106 (77%)	103 (67%)	
Sometimes	82 (17%)	28 (15%)	22 (16%)	32 (21%)	
Several times a week	12 (3%)	5 (3%)	2 (1%)	5 (3%)	< 0.001
Once a day	36 (8%)	22 (12%)	7 (5%)	7 (5%)	
Several times a day	11 (2%)	4 (2%)	1 (1%)	6 (4%)	
**Sugary drinks**	476	184	139	153	
Never	117 (25%)	36 (20%)	24 (17%)	57 (37%)	
Sometimes	180 (38%)	71 (39%)	52 (38%)	57 (37%)	
Several times a week	72 (15%)	34 (18%)	20 (14%)	18 (12%)	< 0.001
Once a day	62 (13%)	30 (16%)	21 (15%)	11 (7%)	
Several times a day	45 (9%)	13 (7%)	22 (16%)	10 (7%)	
**Alcohol**	529	224	152	153	
Never	261 (49%)	140 (62%)	76 (50%)	45 (29%)	
Sometimes	165 (31%)	52 (23%)	50 (33%)	63 (41%)	
Several times a week	73 (14%)	26 (12%)	20 (13%)	27 (18%)	< 0.001
Once a day	23 (5%)	5 (2%)	5 (3%)	13 (9%)	
Several times a day	7 (1%)	1 (1%)	1 (1%)	5 (3%)	
