# Supplementary material for: Systemic Periodontal Risk Score Using an Innovative Machine Learning Strategy: An Observational Study
Source: J Pers Med. 2022 Feb 4;12(2):217. doi: 10.3390/jpm12020217 (PMC8879877; doi:10.3390/jpm12020217)
Supplement: Supplementary file 1 [file jpm-12-00217-s001.zip › Table S2.pdf]

Table S2. Performance of the Multilayer Perceptron model for each category of periodontal health to predict.

	**Precision**	**Recall**	**Specificity**	**F1-Score**
CPITN 0	0.70	0.78	0.70	0.74
CPITN 1-2	0.50	0.23	0.90	0.32
CPITN 3-4	0.57	0.83	0.82	0.68
Average	0.61	0.63	0.78	0.60
