# Supplementary figures and images for: Systemic Periodontal Risk Score Using an Innovative Machine Learning Strategy: An Observational Study
Source: J Pers Med. 2022 Feb 4;12(2):217. doi: 10.3390/jpm12020217 (PMC8879877; doi:10.3390/jpm12020217)

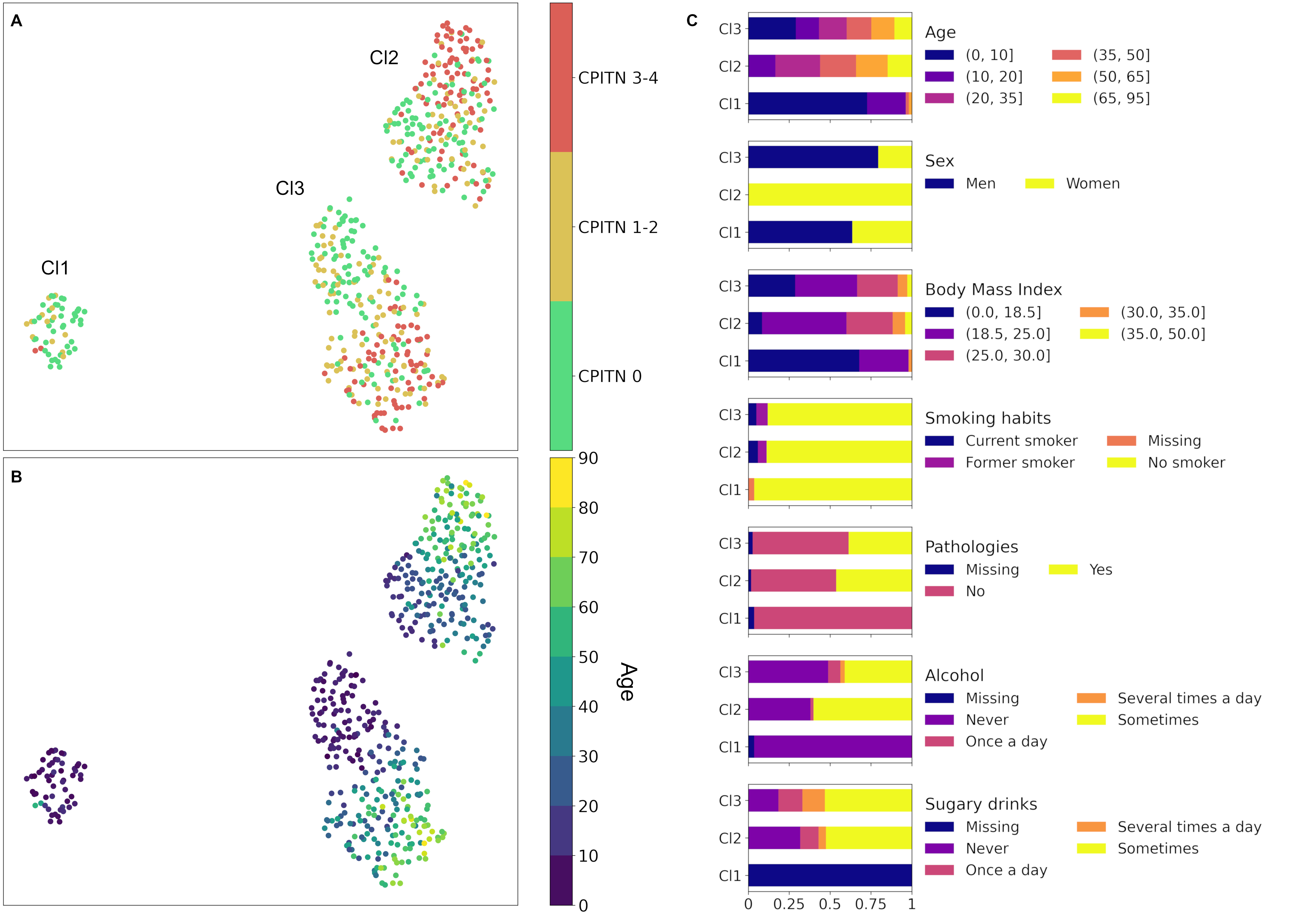

Supplement: Supplementary file 1 [file jpm-12-00217-s001.zip › Figure S2.png]

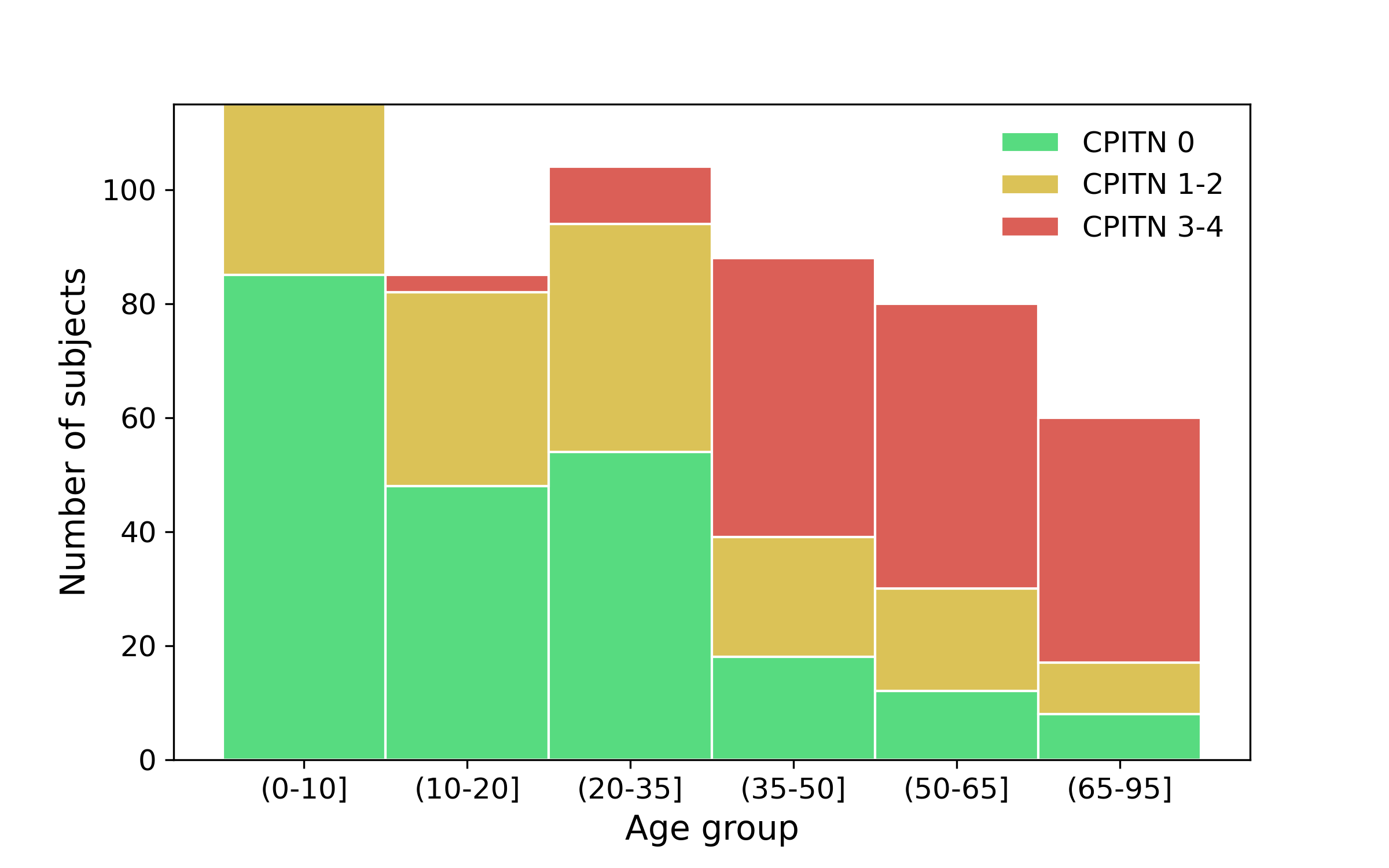

Supplement: Supplementary file 1 [file jpm-12-00217-s001.zip › FigureS1.tif]
